# Mechanical ventilation of acute respiratory distress syndrome

**DOI:** 10.1186/s40560-015-0091-6

**Published:** 2015-05-29

**Authors:** Ryoichi Ochiai

**Affiliations:** Department of Anesthesiology, School of Medicine, Toho University, 6-11-1, Oomori-nishi, Oota-city, Tokyo 143-8541 Japan

**Keywords:** Acute respiratory distress syndrome, Ventilator-induced lung injury, Electrical impedance tomography, Lung protective strategy, Prone positioning, Gravitational effect, Baby lung

## Abstract

Acute respiratory distress syndrome (ARDS) has been intensively and continuously studied in various settings, but its mortality is still as high as 30–40 %. For the last 20 years, lung protective strategy has become a standard care for ARDS, but we still do not know the best way to ventilate patients with ARDS. Tidal volume itself does not seem to have an important role to develop ventilator-induced lung injury (VILI), but the driving pressure, which is inspiratory plateau pressure—PEEP, is the most important to predict and affect the outcome of ARDS, though there is no safe limit for the driving pressure. There is so much controversy regarding what the best PEEP is, whether collapsed lung should be recruited, and what parameters should be measured and evaluated to improve the outcome of ARDS. Since the mechanical ventilation for patients with respiratory failure, including ARDS, is a standard care, we need more dynamic and regional information of ventilation and pulmonary circulation in the injured lungs to evaluate the efficacy of new type of treatment strategy. In addition to the CT scanning of the lung as the gold standard of evaluation, the electrical impedance tomography (EIT) of the lung has been clinically available to provide such information non-invasively and at the bedside. Various parameters have been tested to evaluate the homogeneity of regional ventilation, and EIT could provide us with the information of ventilator settings to minimize VILI.

## Introduction

Acute respiratory distress syndrome (ARDS) was first introduced by Dr. Ashbaugh in 1969 and re-defined as Berlin definition in 2012 as acute respiratory failure in terms of acute onset, hypoxia, diffuse infiltrates on chest X-ray, and absence of cardiac failure, or pulmonary edema due to cardiac origin [1, 2]. The severity of ARDS is solely dependent on the oxygenation failure, expressed as PaO_2_/F_I_O_2_ ratio of 100, 200, and 300 mmHg as severe, moderate, and mild, respectively. Since the introduction of disease entity almost 50 years ago, the mortality has been slightly but consistently improved, but the survival rate is still as low as 70 %.

The reason for such low surviving rate of ARDS might be due to the lack of knowledge and evidence regarding the respiratory anatomy and physiology in normal and ARDS patients as well as the pathological process of ARDS.

We have to recognize what we have already elucidated about the physiological changes in ARDS and understand the pathophysiology of ARDS in order to improve its outcome.

## Review

### The outcome of ARDS

Until the Berlin definition was established [2], the definition of ARDS was somehow ambiguous because of the overlapped criteria of acute lung injury (ALI) and ARDS in terms of the level of hypoxia. By the new definition, ALI could be considered as ARDS of all severity, PaO2/FIO2 <300 mmHg. In the 1980s, the mortality from ARDS was as high as 60–80 %, and it gradually decreased to 30–40 % in the 2000s [3]. Overall pooled weighted mortality from 1984 to 2006 was 44.3 %, and the major effect appears before the publication of the American-European Consensus Conference (AECC) definition of ALI/ARDS in 1994 [4]. Rubenfeld et al. studied the incidence and outcomes of ALI in 21 hospitals in Washington from 1999 through 2000, including 1113 patients on the mechanical ventilation during this period [5]. The crude incidence rate of ALI was 78.9 per 100,000 person-years, and the age-adjusted incidence was 86.2 per 100,000 person-years. The in-hospital mortality was 38.5 % and increased with age from 24 % for patients 15 through 19 years of age to 60 % for those 85 years of age or older. In this population, it is quite interesting that almost 90 % of those patients with ALI were involved with sepsis.

Such high mortality from ARDS might be associated with various factors other than respiratory failure. Some studies reported that the mortality is more commonly related to the development of sepsis and multiple organ failure (MOF) and others that it is related with severity of respiratory failure. Ferring et al. studied the clinical and biological data to elucidate what makes the mortality from ARDS much worse [6]. Over a 2-year period in his ICU, 129 patients were treated for ARDS, defined as PaO2/FIO2 <200 mmHg. The overall mortality rate was 52 %. The primary cause of death was sepsis and MOF (49 %), followed by refractory hypoxia (16 %), cardiac failure or arrhythmias (15 %), neurological failure (10 %), and other causes (8 %). The mortality was related to age and degree of organ failure. In addition, mortality was higher in septic patients than in non-septic patients. Although there has been a report of high incidence of refractory hypoxia as a cause of death by ARDS [7], sepsis and MOF is the leading cause of death in patients with ARDS, and any treatment which can prevent developing sepsis and MOF should be conducted, which is the goal of lung protective strategy [8, 9].

In conclusion, almost 50 years after the introduction of ARDS by Ashbaugh, the mortality is still 30 to 40 %, and such high mortality could be associated with the concomitant development of sepsis and MOF. In order to improve the outcome of ARDS, we have to consider the strategy to reduce the incidence of sepsis and MOF.

### Lung protective ventilation strategies

The first report was by Amato and his coworkers that the protective ventilation strategy with small tidal volume in the patients with ARDS resulted in the better outcome, when compared with those with larger tidal volume, published in 1998 [10]. Until now, there have been six RCTs done to compare the mortality between the groups with smaller tidal volume and with larger tidal volume [10–15]. Those clinical studies are summarized in Table 1.

**Table 1:** Summary of six randomized control trials to compare the outcome of treatment between larger vs. smaller tidal volumes of mechanical ventilation in the patients with ARDS [10–15]

		Author (journal)					
		Amato (NEJM 1998) [10]	ARDSNet (NEJM 2000) [11]	Brochard (AJRCCM 1998) [12]	Brower (CCM 1999) [13]	Stewart (NEJM 1998) [14]	Villar (NEJM 2006) [15]
Control	*n*	24	429	58	26	60	45
	*V*_T_ (ml/kg)	763 (26)^a^	11.8 (0.8)	10.3 (1.7)	10.2 (0.1)	10.7 (1.4)	10.2 (1.2)
	PEEP (cm H_2_O)	6.9 (0.8)	8.6 (3.6)	10.7 (2.3)	8.8 (0.8)	7.2 (3.3)	9.0 (2.7)
	Plateau P. (cm H_2_O)	34.4 (1.9)	33 (9)	31.7 (6.6)	30.6 (0.8)	26.8 (6.7)	32.6 (6.2)
	Mortality (%)	71.0	39.8	37.9	50.0	47.0	53.3
	Pneumonia (%)	63.0	36.0		40.0	48.3	29.0
	Sepsis (%)	79.0	26.0		20.0	35.0	26.7
Protective	*n*	29	432	58	26	60	50
	*V*_T_ (ml/kg)	362 (11)^a^	6.2 (0.9)	7.1 (1.3)	7.3 (0.1)	7.0 (0.7)	7.3 (0.9)
	PEEP (cm H_2_O)	16.3 (0.7)	9.4 (3.6)	10.7 (2.9)	9.8 (0.8)	8.6 (3.0)	14.1 (2.8)
	Plateau P. (cm H_2_O)	31.8 (1.4)	25 (7)	25.7 (5.0)	24.9 (0.8)	22.3 (5.4)	30.6 (6.0)
	Mortality (%)	38.0*	31.0*	46.6	46.0	50.0	32.0*
	Pneumonia (%)	52.0	33.0		70.0	35.0	32.0
	Sepsis (%)	86.0	27.0		20.0	43.3	28.0

Numbers in parentheses are standard deviations

*NEJM* New England Journal of Medicine, *AJRCCM* American Journal of Respiratory and Critical Care Medicine, *CCM* Critical Care Medicine

*p <0.05, compared with control

^a^ml

In these studies, it is quite clear that those patients with ARDS had an extremely wide variety of back grounds in terms of tidal volume, PEEP, inspiratory plateau pressure, and concomitant incidence of sepsis or pneumonia. Indeed, the mortality in control group of the studies by Amato and Villar was 71 and 53 %, respectively, and seems extremely higher than the mortality of ordinary care of 30 to 40 %, which is reported elsewhere [5]. It is clear that the range of ventilatory parameters was overlapped among the groups, and thus the direct comparison is statistically difficult. In 2007 and 2013, the systematic reviews listed those six clinical studies and concluded that the lower tidal volume and inspiratory plateau pressure less than or equal to 31 cm H_2_O significantly reduced the mortality at day 28, hospital mortality, and morbidity [16, 17].

Eichacker et al. presented a meta-analysis of the first five randomized controlled trials of lung protective strategy [10–14], and proposed that in the two beneficial trials, the differences in mortality appear attributable to the increased mortality in the control arms as opposed to benefit in the low tidal volume arms, most likely due to the extremely higher plateau pressure in the control group of two beneficial groups [18].

Amato et al. finally summarized the clinical effects of ventilatory components on the outcome of patients with ARDS [19]. Mechanical-ventilation strategies that use lower end-inspiratory (plateau) airway pressures, lower tidal volumes (*V*_T_), and higher positive end-expiratory pressure (PEEP) can improve survival in patients with ARDS, but the relative importance of each component has not been clear. Each component is closely related with each other. Because respiratory-system compliance (*C*_RS_) is strongly related to the volume of aerated remaining lung (termed functional lung size), they hypothesized that the driving pressure (Δ*P* = *V*_T_/*C*_RS_), which is tidal volume normalized in relation to *C*_RS_ but not by body weight, would be a better predictor of survival than *V*_T_ or PEEP in patients with ARDS.

They analyzed individual data from 3562 patients with ARDS enrolled in nine previously reported randomized trials in order to examine Δ*P* as an independent variable associated with survival. As a result, two baseline variables (risk according to APACHE or SAPS and arterial pH) and two ventilator variables (F_I_O_2_ and Δ*P*) were significantly associated with survival after multivariate adjustment. Higher Δ*P* predicted lower survival consistently across trials (*P* = 0.13 for heterogeneity).

**Fig. 1:**
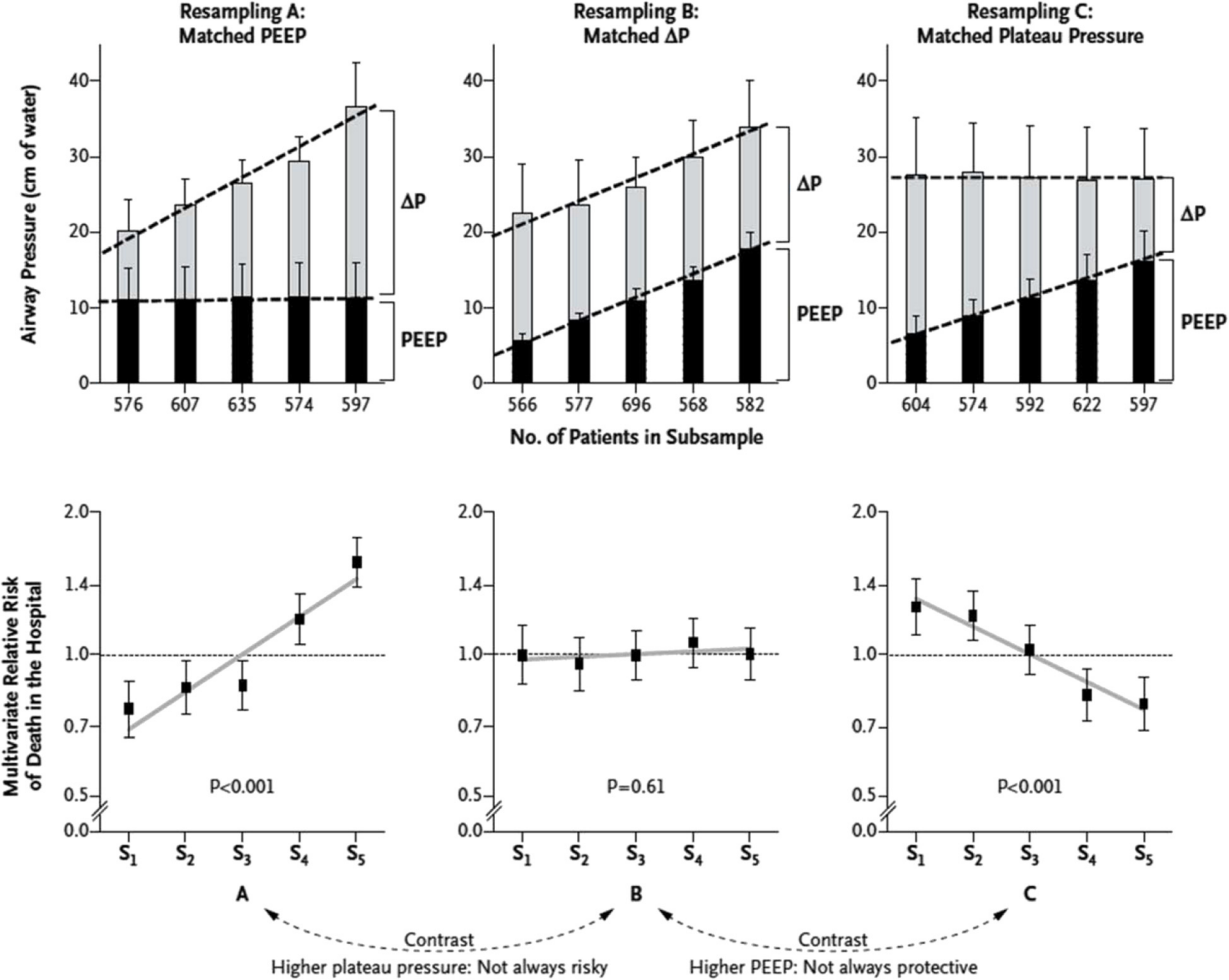
Relative risk of death in the hospital across relevant subsamples after multivariate adjustment—survival effect of ventilation pressures [19]. The *upper stacked-bar diagrams* illustrate the mean values for PEEP, inspiratory plateau pressure, and driving pressure (ΔP) observed in each subsample. The *error bars* represent 1 standard deviation. At the *bottom*, the respective relative risks for death in the hospital are shown, calculated for each subsample after multivariate adjustment (at the patient level) for the five covariates (trial, age, risk of death according to the Acute Physiology and Chronic Health Evaluation (APACHE) or Simplified Acute Physiology Score (SAPS), arterial pH at entry, and Pao2:Fio2 at entry) specified in model 1. *Error bars* represent 95 % confidence intervals. A relative risk of 1 represents the mean risk of the pooled population, which had an adjusted survival rate of 68 % at 60 days. With permission from the publisher

Figure 1 shows that in the pooled sample (including 3562 patients), higher plateau pressures were observed in patients with higher Δ*P* or higher PEEP, but with different consequences (resampling A vs. B): higher mortality was noted only when higher plateau pressures were observed in patients with higher Δ*P*s. Similarly, the protective effects of higher PEEP were noted only when there were associated decreases in Δ*P* (resampling B vs. C). In addition, at constant levels of plateau pressure, *V*_T_ was a strong predictor of survival when normalized to *C*_RS_ (i.e., Δ*P*) but not when normalized to predicted body weight.

### Tidal volume and other parameters

As mentioned above, the outcome of ARDS is dependent on the involvement of MOF other than respiratory failure. There is evidence that the way of mechanical ventilation has a significant effect on the cause of MOF. Indeed, unfortunately, there are still many who believed that the tidal volume of 6 ml/kg of predicted normal weight is a safe method to ventilate the patient with ARDS, but the essence of lung protective strategy is clearly demonstrated above and is to protect the lesion where the normal lung mechanics is maintained, from overstretch in terms of computed tomography (CT) numbers.

In the lungs which suffered from ARDS, the common pathophysiology is systematic inflammation and resultant pulmonary edema. The lungs of ARDS are wet and heavy, and the part of the lungs which seems normal and ready to accept the tidal ventilation is quite limited and probably dependent on the percentage of aeration of the diseased lungs. Mechanical ventilation, which is a standard therapy to maintain adequate gas exchange during ARDS, may lead to the acceleration of inflammatory process and may augment a pulmonary damage (ventilator-induced lung injury (VILI)). Indeed, analysis of CT images of patients with ARDS has demonstrated a non-homogenous distribution of pulmonary changes, such as hyperinflated, normally aerated, poorly aerated, and non-aerated compartments, according to the CT numbers (Hounsfield unit) [20, 21].

Gattinoni and coworkers provided the direct visual and biochemical evidence that the same tidal volume means different in the patients with different lung structures and alterations due to ARDS [22]. The adult patients with early ARDS were studied and ventilated with ARDSnet protective ventilator strategy. The patients studied were divided into two groups: the “more protected” group, where the tidal hyperinflation was less than 10 %, and the “less protected” group, with greater than 60 % of hyperinflation, while all the patients were ventilated with the same tidal volume of 6 ml/kg of predicted body weight. The less protected group is characterized by more hypoxic, higher plateau pressure, higher PEEP, and same static lung compliance. Simultaneously with chest CT scanning, the bronchoalveolar lavage was conducted and inflammatory cytokines, such as IL-6, IL-1β, IL-1ra, IL-8, and TNF-α were measured. In the “less protected” group, significantly higher concentrations of those cytokines were confirmed in the lavage fluid. This study clearly demonstrated that the ARDSnet lung protective strategy may not be protective of all patients with ARDS, and in the patients with heavier lungs, a larger non-aerated dependent compartment and less normally aerated compartment, VILI was induced because of the hyperinflation of the small normal lung despite of lowering tidal volume to 6 ml/kg and lowering plateau pressure less than 30 cm H_2_O. And, an insufficient level of PEEP may cause tidal recruitment/derecruitment of the consolidated/poorly aerated region and can expose these regions to shear stress, increasing cytokines from lungs, leading to the MOF in remote organs [22, 23].

Much smaller tidal volume was challenged by using extracorporeal approach. In severe ARDS, one of the alternative treatments other than mechanical ventilation is extracorporeal membrane oxygenation (ECMO), and its clinical significance has been proven [24, 25]. But, ECMO is still a highly invasive treatment with significant risk and complications, with the mortality of 50–60 % [24, 25], which is highly dependent on the pre-ECMO parameters, presented as PRESERVE score [26]. It is assumed less invasive to use arteriovenous extracorporeal membrane carbon dioxide removal (AVECCO2R) than ECMO, but evidence is highly limited on the efficacy of AVECCO2R. Bein and his coworkers compared the two groups of ARDS on ventilator-free days and mortality, one with a low tidal volume strategy (*V*_T_ ~3 m/kg-predicted body weight) using pumpless extracorporeal lung assist (AVECCO2R) and another with ARDSNet strategy (~6 ml/kg) without AVECCO2R [27]. There was no significant difference in ventilator-free-day and mortality between the groups. Again, since this study did not adjust the tidal volume by static compliance, but only by body weight, it is clear the tidal volume itself has a limited importance in the treatment of ARDS, and the amount of aerated areas should be considered.

### VILI and hyperinflation/overstretch of the lung

Lung protective strategy and its success are dependent on the amount of aerated area in the ARDS lungs, and normally aerated region is highly variable among the patients and its severity of inflammation. The concept of baby lung was first introduced in middle 1980s [28], and it was presented that the respiratory-system compliance was well correlated only with the amount of normally aerated tissue. Gattinoni discovered that the ARDS lung is not stiff, but small, and the specific compliance of the residual inflated lung is nearly normal, as indicated by the specific tissue compliance [29, 30]. Baby lung is located primarily in the non-dependent lung regions, but its position in the lung is likely to be dependent on the gravitational effect on the lung structure, since the high density in the dorsal regions in the supine position redistributes to the ventral regions in prone position [31]. The gravitational effect on the regional distribution of ventilation and pulmonary circulation should be clarified in both healthy and injured lungs to understand the disease process and treatment strategy of ARDS.

### Gravitational effect on the ventilation and pulmonary circulation

In order to understand the management of ARDS, we have to understand the regional differences in ventilation and perfusion of the lung. Indeed, several current textbooks state that gravity has a predominant effect on pulmonary regional blood flow, but in some other textbooks that the recent research has shown that factors such as the basic anatomical structure of the pulmonary vessels and airways may be as important as gravity in determining regional distribution of blood flow and ventilation.

### Gravity and prone position in the healthy lungs

One of the most popular findings of the effect of gravity on respiratory system was introduced by West in 1964, and his result was that the lung is categorized in three distinctive zones: zones 1–3, dependent on the relationship among pulmonary arterial and venous pressures, and alveolar (airway) pressure [32]. In zone 1, alveolar pressure exceeds vascular pressures, resulting in vascular collapse. In zones 2 and 3, vascular pressure exceeds alveolar pressure, leading to the more blood flow at the gravitational gradient. This zoning is based on his unique experiment using microsphere technique in isolated canine lungs [32]. The lung was isolated and suspended (alveolar pressure 0 cm H_2_O) in the negative pressure chamber (−10 cm H_2_O), and pulmonary circulation (mean pulmonary arterial pressure of 32 mmHg) was achieved by the arterial blood supply from another animal. The radioactivity of injected Xe was counted to calculate the regional pulmonary blood flow.

In reality, the lungs are inside the thoracic cavity, and its own weight and gravitational effects influence lung structure and its shape. The three zones of lung perfusion by West do not include those physiological and anatomical factors, and cannot be applied to the normal as well as diseased lungs; thus, we have to re-evaluate the gravitational effects on the ARDS-lungs.

Various studies have been done, and one of the most interesting studies was done by Petersson and coworkers, using single-photon-emission computed tomography (SPECT) in the healthy volunteers [33].

Their study evaluated the effect of gravity on the pulmonary circulation. To make the gravitational effect more clear, they measured during high gravity condition using centrifugation up to 3G, and Tc-labeled macroaggregates of albumin (MAA) were injected during centrifugation both in supine and prone positions to measure regional blood flow by using SPECT.

**Fig. 2:**
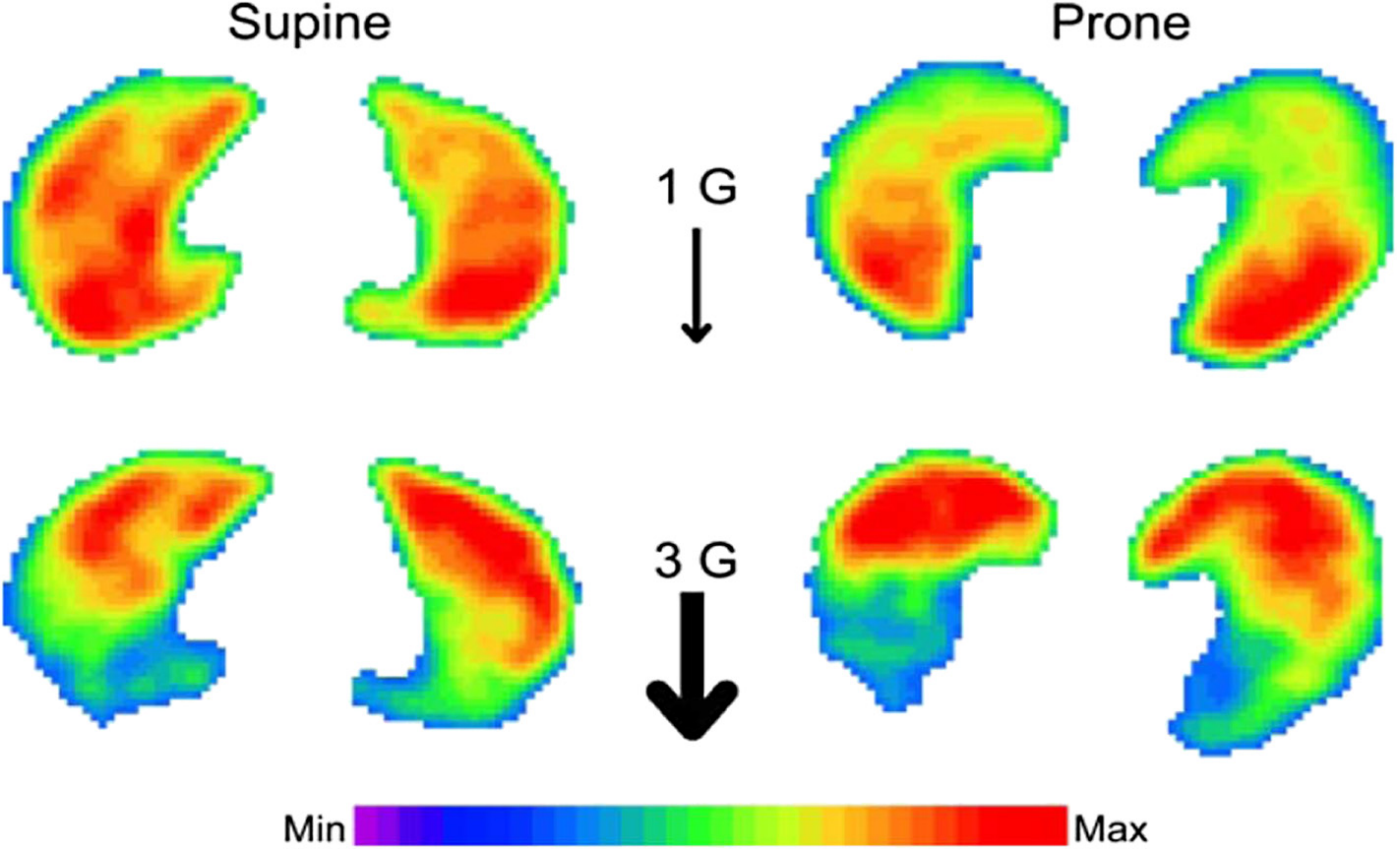
The distribution of pulmonary blood flow in supine or prone position under normal gravity or hypergravity of 3G [33]. SPECT images representing blood flow distribution within a transverse lung section for all conditions in subject 4. Coloring is according to a relative scale for each image. With permission from the publisher

As shown in Fig. 2, during normal gravity, in supine position, blood flow is evenly distributed both in dependent and non-dependent areas, but in prone position, more blood flow was found in the dependent area. On the other hand, during hypergravity, redistribution of blood flow from dependent to non-dependent lung regions implies an increase in vascular resistance in dependent regions either through an increase in vascular tone, e.g., hypoxic vasoconstriction, or through mechanical factors. It is likely that the weight of the lung itself might squeeze out the blood flow from the dependent area to non-dependent area, and most of the blood flow is measured in the non-dependent area in both supine and prone positions during hypergravity. It could be speculated that even during normal gravity, the density of the lung is much heavier in the dependent area even in normal lungs; the blood flow should be shifted to non-dependent area, but some control mechanism might change the distribution. One could easily imagine what will happen in the patient with ARDS, which is most popular with pulmonary edema and inflammation, resulting in the “heavy lung.” Higher pulmonary tissue density will act as that of hypergravity, thus compressing the pulmonary parenchyma of the dependent lung.

Such mechanism was also demonstrated by Remeika and his coworkers, by using the same technique, SPECT and 99mcTc-MMA injections; the distribution of blood flow was measured before and after the injection of NOS inhibitor (L-NMMA) [34]. It was shown that the blood flow distribution was shifted from dependent area to non-dependent area after the inhibition of NO production by using L-NMMA. Even in normal gravity, the regional pulmonary blood flow in the dependent regions is shifted to non-dependent region due to the weight of the lung itself as well as mediastinal organs. Nitric oxide might be one of the factors to control its homogenous distribution of pulmonary blood flow in normal healthy humans.

### What happens to the ventilation distribution during supine as well as prone position?

As mentioned above, the intensive evaluation has been done to look at the gravitational effect on the regional pulmonary blood flow, and regional ventilation, and it has been also evaluated by means of various interventions. One of those was done in the healthy volunteers, who were anesthetized with propofol and mechanically ventilated, and regional ventilation and pulmonary perfusion were evaluated using the same SPECT technique as previous studies [35]. They measured regional pulmonary perfusion and ventilation and calculated ventilation/perfusion ratio (V/Q ratio) at 21 gravitational regions from ventral to dorsal orientation. They found a more homogenous V/Q ratio in the lungs in prone position than in supine position.

Those SPECT studies in healthy volunteers indicate very important findings; the lungs in the thorax is affected by the gravitational effect, the weight of the lung itself would squeeze the regional blood flow from dependent region to non-dependent region, the distribution of blood flow would be adjusted by the production of NO in the dependent region to maintain the homogenous V/Q ratio in the lungs, and V/Q ratio would be more homogenous in prone position than in supine position. Prone position might be more physiologically appropriate than supine in human population.

We still do not know whether such adjustment to obtain a more homogenous distribution of regional pulmonary blood flow and ventilation is also true in patients with ARDS, thus, the goal of the lung protective strategy would be better homogeneity of regional ventilation and perfusion in those suffered lungs.

There is an important suggestion that the gravity is not the only factor to control the distribution of pulmonary blood flow and ventilation, because the blood flow at the same vertical height (iso-heights) was not uniformly distributed [36–38]. Regional ventilation was also measured, and a wide variety of distribution, independent from gravity was found [39]. Those findings suggest that under conditions of constant cardiac output and perfusion pressure, variations in blood flow arise from the basic architecture of the pulmonary vessels, and the same mechanism could determine the regional distribution of ventilation [40, 41]. In patients with ARDS, their lung structure is highly affected by serious inflammation and pulmonary edema, and the increased weight would act as hypergravity to squeeze the blood flow as well as ventilation out of the dependent area to the non-dependent region. And the prone positioning is preferable for those patients, since the distribution of pulmonary perfusion and ventilation is more homogenous than in the supine position even in the diseased lungs.

### Baby lungs and alveolar recruitment

As mentioned above, the ARDS lung is characterized by the small-aerated region, called baby lung. The damage to the alveolar-capillary membrane leads to high-permeability edema with wash-out or dilution of the surfactant and inactivation of the surfactant by plasma components, such as fibrin, albumin, globulin and hemoglobin, and cell membrane lipids [42, 43]. The large and injurious tidal volume is one of the factors, which disturb pulmonary surfactant. Isolated rat lungs were mechanically ventilated with large tidal volume of 20 ml/kg without PEEP, and morphometric analysis was done to quantify the components of surfactants such as tubular myelin, lamellar body, and multilamellar structure [44]. The amount of those pulmonary surfactants was highly reduced, and minimum surface tension increased while lung compliance was decreased in the injurious group, compared with control group. Thus, ARDS is associated with systematic and lung inflammation, and the reduction of pulmonary surfactant will increase the surface tension of the alveoli and also increase the shear stress among the affected alveoli. Such disease process may set a question whether we have to open the atelectatic lung region. If we could open the atelectasis during the mechanical ventilation and keep it open, it will reduce the stress-induced-inflammation and improve the gas exchange, especially oxygenation. However, there has been not enough evidence whether we have to open up the lung in order to improve the outcome of ARDS.

There have been issues regarding the way of mechanical ventilation of patients with ARDS, and relatively higher PEEP may keep the alveoli open at end-expiration, thus preventing atelectrauma and biotrauma [45, 46]. Various meta-analysis and systematic reviews to investigate the role of PEEP for ARDS have resulted in the inconsistent conclusions. It could be because the disease process of each patient was different and the level of PEEP, which was needed to keep the alveoli open, was not properly evaluated nor known. Recruitment maneuvers (RM) are often preformed to increase the volume of aerated lungs, thereby improving the gas exchange. Sustained CPAP as high as 40 cm H_2_O, periodic sighs, step-wise increase in PEEP, and inspiratory pressure have been attempted. It is all dependent on the amount of aerated region of the lungs to determine the VILI, while the RM might worsen tidal hyperinflation, with over-distension of compliant, or normal, part of the lung tissue, predisposing them to VILI [47, 48]. Despite an improvement in oxygenation, clinical trials have not found a survival benefit, and there is insufficient evidence for routine use of RMs at this stage [49–51].

One of such approaches was to evaluate the effect of PEEP to recruit the ARDS-model-lungs on the best compromise between mechanical stress and lung aeration in oleic acid-induced lung injury [52]. In this study, the adjustment of PEEP to avoid both alveolar derecruitment and hyperinflation was evaluated by CT scanning by measuring the distribution of lung aeration.

In conclusion, PEEP at which the highest respiratory-system compliance occurred, obtained by descending PEEP titration, corresponded to the largest amount of normally aerated areas, with the least amount of collapsed and hyperinflated areas. The institution of higher levels of PEEP reduced both compliance and poorly aerated areas but increased hyperinflated areas. The lower PEEP level consistently enhanced poorly or non-aerated areas as well as tidal re-aeration, with the reduction in compliance. Hence, monitoring respiratory mechanics during a PEEP titration procedure may be a useful adjunct to optimize lung aeration.

Thus, the optimal setting of mechanical ventilation has been challenged by means of chest CT scanning [53, 54], but such approach might not be practical for the patients on mechanical ventilation in the ICU. Less invasive and continuous monitoring of regional ventilation is desirable, since the ventilation settings are of such high importance to improve the outcome of patients with ARDS.

### Electrical impedance tomography, a new type of monitoring in the future

By recruiting the collapsed lungs, we try to open up the lungs and keep it open to improve gas exchange and to reduce the stress by mechanical ventilation. The ideal goal is to minimize the mechanical-ventilation-induced stress on the lungs, in order to minimize the part of the lungs with hyperinflation as well as collapse. This is the best compromise of mechanical ventilation, because the airway pressures, such as inspiratory plateau pressure and PEEP level, are common to all the airway and alveoli. It is a common way to assess the appropriateness of ventilation by CT scanning data, since CT is regarded as the gold standard to assess the effect of a recruitment maneuver and PEEP level applied on the aeration of the atelectatic lung [53, 54]. However, the obvious disadvantage of repeated CT scans, such as transportation-related risks and excessive radiation exposure, reduces the application of CT as a tool for assessment of recruitment.

On the other hand, electrical impedance tomography (EIT) is a real-time monitoring device, which has proven to correlate well with CT for assessment of changes in gas volume and tidal volume [55–57]. Several EIT parameters have been developed to collect more data on ventilation distribution in order to optimize ventilator settings [58–60]. Typical parameters used to describe the homogeneity of ventilation in the lungs are regional ventilation delay (RVD) [61, 62], intra-tidal gas distribution (ITV) and its index (ITVI) [61], center of ventilation (COV) [59], and global inhomogeneity index (GII) [63]. Blankman and coworkers studied a decremental PEEP trial in 12 post-cardiac surgery patients, and at each PEEP step, those EIT parameters were measured and evaluated [64]. They examined whether one specific EIT parameter is able to describe the optimal PEEP level at the bedside. In those postoperative patients, ITV index was comparable with dynamic compliance to indicated optimal PEEP level, minimizing overdistention in the non-dependent lung and lung collapse in the dependent lung.

EIT could provide us with a new type of monitoring the regional distribution of ventilation non-invasively and continuously at the bedside. However, extensive clinical studies are needed to elucidate such information could lead to better outcome in patients with ARDS.

## Conclusions

The pathophysiology of ARDS has been intensively and continuously studied in both clinical and experimental settings for the last 50 years, but still the mortality rate for ARDS is as high as 30–40 %. The lung protective ventilation has become the standard treatment strategy for the patients with ARDS. It has been clearly demonstrated that the driving pressure could be the ventilatory parameter, which significantly predicts and affects the outcome, based on the pooled data by using a statistical tool known as multilevel mediation analysis. The lower the driving pressure, the better the outcome. It is essential to prove that this statistical finding is true for patients with ARDS in clinical settings. Moreover, although in that article, the level of PEEP does not affect the outcome, we still have to elucidate how to determine the best level of PEEP in order to obtain homogenous gas distribution, thus improving oxygenation and lung injury.

Prone positioning seems physiologically correct in terms of better gas exchange, but it should be considered with monitoring the regional distribution of both ventilation and pulmonary perfusion. And gravitational effects on both ventilation and pulmonary perfusion in ARDS should be clarified.

Mechanical ventilation could be harmful for the healthy as well as injured lungs by an inappropriate setting of the ventilator, but mechanical ventilation is still and will be a standard care for patients with ARDS even after the introduction of ECMO. For a better outcome of ARDS, there are various questions of mechanical ventilation to be solved, such as management of spontaneous breathing, use of neuromuscular blocking agents, and clinical significance of transpulmonary-pressure, which will provide a new approach to the settings of mechanical ventilation. All the answers to these things are too fascinating to wait for.

## Abbreviations

ARDS: acute respiratory distress syndrome
ALI: acute lung injury
RCT: randomized controlled study
RR: relative risk
APACHE: Acute Physiology and Chronic Health Evaluation
VILI: ventilator-induced lung injury
CT: computed tomogramphy
ECMO: extracorporeal membrane oxygenation
